# Molecular Profile of Intrahepatic Cholangiocarcinoma

**DOI:** 10.3390/ijms25010461

**Published:** 2023-12-29

**Authors:** Wellington Andraus, Francisco Tustumi, José Donizeti de Meira Junior, Rafael Soares Nunes Pinheiro, Daniel Reis Waisberg, Liliana Ducatti Lopes, Rubens Macedo Arantes, Vinicius Rocha Santos, Rodrigo Bronze de Martino, Luiz Augusto Carneiro D’Albuquerque

**Affiliations:** Department of Gastroenterology, Transplantation Unit, Universidade de São Paulo, São Paulo 05403-000, Brazil

**Keywords:** tumor biomarkers, cholangiocarcinoma, liver neoplasms

## Abstract

Intrahepatic cholangiocarcinoma (ICC) is a relatively uncommon but highly aggressive primary liver cancer that originates within the liver. The aim of this study is to review the molecular profile of intrahepatic cholangiocarcinoma and its implications for prognostication and decision-making. This comprehensive characterization of ICC tumors sheds light on the disease’s underlying biology and offers a foundation for more personalized treatment strategies. This is a narrative review of the prognostic and therapeutic role of the molecular profile of ICC. Knowing the molecular profile of tumors helps determine prognosis and support certain target therapies. The molecular panel in ICC helps to select patients for specific therapies, predict treatment responses, and monitor treatment responses. Precision medicine in ICC can promote improvement in prognosis and reduce unnecessary toxicity and might have a significant role in the management of ICC in the following years. The main mutations in ICC are in *tumor protein p53* (*TP53*), *Kirsten rat sarcoma virus* (*KRAS*), *isocitrate dehydrogenase 1* (*IDH1*), and *AT-rich interactive domain-containing protein 1A* (*ARID1A*). The rate of mutations varies significantly for each population. Targeting *TP53* and *KRAS* is challenging due to the natural characteristics of these genes. Different stages of clinical studies have shown encouraging results with inhibitors of mutated *IDH1* and target therapy for *ARID1A* downstream effectors. *Fibroblast growth factor receptor 2* (*FGFR2*) fusions are an important target in patients with ICC. Immune checkpoint blockade can be applied to a small percentage of ICC patients. Molecular profiling in ICC represents a groundbreaking approach to understanding and managing this complex liver cancer. As our comprehension of ICC’s molecular intricacies continues to expand, so does the potential for offering patients more precise and effective treatments. The integration of molecular profiling into clinical practice signifies the dawn of a new era in ICC care, emphasizing personalized medicine in the ongoing battle against this malignancy.

## 1. Introduction

Cholangiocarcinoma is the second-most common primary liver malignancy, following hepatocellular carcinoma [1]. It is categorized based on its anatomical location within the biliary tree as perihilar, distal, or intrahepatic cholangiocarcinoma. Perihilar cholangiocarcinoma accounts for 50–60% of cases and is defined as the one positioned proximal to the cystic duct’s origin and distal to the second-order bile ducts. Meanwhile, distal cholangiocarcinoma constitutes 20–30% of cases, manifesting distal to the cystic duct’s origin [1,2].

Intrahepatic cholangiocarcinoma (ICC) is a relatively uncommon but highly aggressive primary liver cancer defined as that occurring proximal to the second-order bile ducts and corresponding to 10% of all cholangiocarcinomas [1,2]. The primary risk factors for ICC include chronic infections, liver cirrhosis, metabolic factors, bile duct disorders, environmental toxins, and genetic factors [3]. The incidence of cholangiocarcinoma has increased since 2001, with a greater increase in ICC than extrahepatic cholangiocarcinoma. In the United States, ICC incidence is 1.99 per 100,000 person-years [4]. The mortality rate ranges from 0.34 to 2.67 per 100,000 person-years for males and females from Western countries [2].

There is notable diversity in its epidemiological patterns across different regions and populations. Geographical variations in ICC incidence are striking, with Southeast Asia being a notable hotspot. In this region, where liver fluke infection is prevalent, chronic parasitic infections, specifically *Opisthorchis viverrini* and *Clonorchis sinensis*, significantly increase the risk of ICC [5,6]. The chronic inflammation induced by these parasites is thought to contribute significantly to the development of ICC. In Western countries, the incidence of ICC has evolved over recent years. There has been a noticeable increase in new ICC diagnoses, often attributed to the growing prevalence of non-alcoholic fatty liver disease (NAFLD) [7]. This condition is closely linked to obesity, metabolic syndrome, and diabetes, which are recognized as key risk factors for ICC development. Liver cirrhosis, often caused by chronic hepatitis B or C infections, excessive alcohol consumption, or NAFLD, is another major risk factor for ICC [8]. Cirrhosis results in chronic liver inflammation and scarring, which can promote carcinogenesis.

Some diseases, such as primary sclerosing cholangitis (PSC) and choledochal cysts, which affect the bile ducts, are also linked to an elevated risk of ICC. Exposure to certain environmental toxins, including thorium dioxide, previously used in certain medical procedures, and certain chemicals in workplaces like the printing and dyeing industries may increase the risk of ICC. Some genetic conditions, like Lynch syndrome and Caroli disease, are also associated with a significant risk of developing ICC [9].

The prognosis for patients diagnosed with intrahepatic cholangiocarcinoma is frequently daunting [10]. A significant challenge is that ICC often remains asymptomatic until it reaches an advanced stage, leading to advanced-stage cancer at diagnosis and diminished treatment options. Furthermore, ICC exhibits early vascular invasion and metastasis, contributing to the poor prognosis. The five-year survival rate for ICC typically falls below 20%. However, it is crucial to acknowledge that prognosis can vary substantially depending on individual patient factors, tumor stage, and molecular characteristics [11].

Recent advances in molecular profiling have revealed distinct molecular subtypes within ICC. These subtypes may be key to understanding prognostic variations and treatment responses. This exciting development offers hope for more personalized prognostic tools and targeted therapeutic approaches tailored to the unique genetic profile of each patient’s tumor [12]. Molecular profiling, as a diagnostic and research tool, plays a pivotal role in deciphering the intricate genetic and molecular alterations that underlie the development and progression of ICC.

Molecular profiling aims to pinpoint unique molecules, including DNA, RNA, and proteins. Numerous methods can be applied for this purpose. Among these methods, conventional tests such as fluorescence in situ hybridization (FISH), immunohistochemistry (IHC), and quantitative polymerase chain reaction (qPCR) are rooted in precision medicine practice. IHC assesses protein expression patterns in ICC by examining the presence and location of specific proteins within the tumor. FISH represents another valuable tool in cancer molecular profiling. FISH aids in detecting chromosomal abnormalities and gene amplifications by visually revealing genetic changes at the chromosomal level. Lastly, qPCR is a technique employed to amplify minute quantities of DNA. This amplification process allows for the precise study of specific DNA segments [13].

More recently, next-generation sequencing (NGS) has taken the lead as the primary tool in molecular profiling. NGS can assess concurrent analysis of a broad range of genomic alterations, not just a single pre-specified gene or protein expression. NGS facilitates the high-throughput sequencing of genomes, allowing the uncovering of genetic mutations, small insertions or deletions (indels), and structural changes within the DNA of the tumor. NGS allows the detection of a wide array of DNA alterations, including mutations, copy number variations, and gene fusions across the entire genome [14]. NGS’s bioinformatics tools analyze the sequencing data to identify instances where sequencing reads span the fusion breakpoint, indicating the fusion event. Additionally, when a genomic region or gene is amplified, NGS generates more sequencing reads that align with that region [15]. This increased read depth signifies amplification. Bioinformatics tools analyze the sequencing data, using specific algorithms to identify regions with higher read coverage than the rest of the genome. Amplifications appear as peaks in read coverage, and the degree of amplification can be quantified based on the extent of the read depth increase. Copy number variations (CNVs), representing deviations from the standard genomic copy number, can also be detected through NGS. NGS assesses read depth and read distribution across the genome. CNVs, like amplifications, lead to changes in read depth for specific genomic regions. NGS captures this information by sequencing the entire genome, producing a profile of read depth. Bioinformatics algorithms then analyze these profiles and pinpoint regions with deviations from the anticipated read depth, revealing the presence of CNVs. Identifying CNVs allows for characterizing the affected genomic regions and genes, contributing to our understanding of their role in diseases.

Molecular profiling in cancer represents a transformative approach that has revolutionized our understanding of the disease and significantly impacted patient care. This cutting-edge technique involves the comprehensive analysis of a tumor’s genetic, molecular, and cellular characteristics. Through the analysis of tumor tissues, molecular profiling aims to identify specific genetic mutations, epigenetic changes, and protein expression patterns unique to each patient’s cancer. By deciphering the intricate genetic mutations, alterations, and biomarkers unique to each patient’s cancer, molecular profiling allows oncologists to tailor treatment strategies with remarkable precision. It helps identify specific targeted therapies, predict treatment responses, and stratify patients into subgroups for clinical trials, enabling more effective and personalized cancer management. As the field of molecular profiling continues to advance, it holds the promise of improving diagnostic accuracy and unlocking new avenues for innovative therapies, ultimately fostering more favorable outcomes for cancer patients [13].

Based on this assumption, the likely future ICC treatment should be based not on histology alone (“one therapy fits all” approach) but instead on personalized medicine, according to each individual’s cholangiocarcinoma tumor characteristics.

The aim of this study is to review the molecular profile of intrahepatic cholangiocarcinoma and its implications for prognostication and decision-making. This comprehensive characterization of ICC tumors sheds light on the disease’s underlying biology and offers a foundation for more personalized treatment strategies.

## 2. Methods

This is a narrative review of the prognostic and therapeutic role of the molecular profile of ICC.

### 2.1. Database Search

A non-systematic search was conducted in PubMed, Embase, Cochrane, Google Scholar, LILACS, and a manual search of references. The search was conducted from the database’s inception to September 2023. The following search terms were used: “molecular panel”, “biomarker”, “precision-medicine”, “target-therapy”, and “intrahepatic cholangiocarcinoma”.

### 2.2. Study Selection

The inclusion criteria were: (a) studies that evaluate patients with a confirmed diagnosis of ICC; (b) studies that evaluate cancer molecular panel; (c) studies that evaluate prognosis, target therapies, and cancer response to therapy; (d) English or Portuguese articles. The exclusion criteria were: (a) case reports, reviews, letters, editorials, and congress abstracts; and (b) full-text unavailability. Two reviewers (F.T. and W.A.) searched and selected the articles using the previously defined eligibility criteria.

### 2.3. Outcomes

The main outcomes evaluated were the prognosis related to the molecular panel, including long-term survival rates, oncologic staging, and response to therapy.

### 2.4. Data Extraction

Two researchers (F.T. and W.A.) extracted the following data: the baseline characteristics of the included studies (study design, year of publication, sex, age, neoadjuvant and adjuvant regimen, surgical therapies) and the outcomes (treatment response, oncologic staging, and prognostic variables).

### 2.5. Data Synthesis

Considering that the studies we included in our analysis showed significant heterogeneity in terms of study design, the clinical condition of patients, their disease stage, and the treatments they received, a qualitative synthesis was performed.

## 3. Results

After study selection, 77 studies [16,17,18,19,20,21,22,23,24,25,26,27,28,29,30,31,32,33,34,35,36,37,38,39,40,41,42,43,44,45,46,47,48,49,50,51,52,53,54,55,56,57,58,59,60,61,62,63,64,65,66,67,68,69,70,71,72,73,74,75,76,77,78,79,80,81,82,83,84,85,86,87,88,89,90,91] were included in this review, composed of pre-clinical studies, different stages of clinical trials, and observational analysis.

### 3.1. Molecular Subtypes in Intrahepatic Cholangiocarcinoma

Our understanding of ICC molecular subtypes is still evolving, and ongoing research may uncover additional subtypes or refine existing classifications. Identifying these subtypes holds promise for tailoring treatment strategies, predicting patient outcomes, and guiding the development of targeted therapies in the future. These subtypes provide insights into the diverse biological underpinnings of ICC and may eventually lead to more targeted and personalized treatment strategies.

At least one gene mutation is found in more than 60% of the biliary tumors, although this rate varies significantly according to the analyzed population [16]. Tomczak et al. [17] evaluated 101 ICC patients who received molecular profiling and matched treatment. Genetic mutations were found in 77% of patients. The most commonly altered genes in tumor tissues were *BRCA1-associated protein 1* (*BAP1*) (23%), *AT-rich interaction domain 1A* (*ARID1A*) (22%), *fibroblast growth factor receptor 2* (*FGFR2*) (22%), *isocitrate dehydrogenase isozyme 1* (*IDH1*) (22%), *cyclin-dependent kinase inhibitor 2A* (*CDKN2A*) (15%), *CDKN2B* (14%), *phosphatidylinositol-4,5-bisphosphate 3-kinase catalytic subunit alpha* (*PIK3CA*) (14%), *TP53* (11%), *ataxia-telangiectasia mutated* (*ATM*) (9%), *IDH2* (9%), *v-raf murine sarcoma viral oncogene homolog B1* (*BRAF*) (7%), *SMARCA4* (7%), and *FGFR3* (5%).

The main ICC gene mutations reported in the somatic mutation database Catalogue of Somatic Mutations in Cancer (COSMIC) (http://www.sanger.ac.uk/cosmic, accessed on 21 December 2023) are *Kirsten rat sarcoma viral oncogene homolog* (*KRAS*), *tumor protein p53* (*TP53*), and *ARID1A*.

Voss et al. [18] found that 24% of their ICC patients had mutations. There were mutations in KRAS, BRAF, epidermal growth factor receptor (EGFR), mesenchymal epithelial transition (MET), neuroblastoma Ras viral oncogene homolog (NRAS), and PIK3CA. These mutations are composed of point mutations, gene amplifications, and changes in chromosome structure that can lead to the formation of fusion proteins.

Weinberg et al. [16] investigated 592 genes in biliary tumors. The most prevalent mutations were in *TP53* (42.7%), *ARID1A* (21.7%), *KRAS* (15.7%), *IDH1* (8.7%), *CDKN2A* (7.8%), *BAP1* (6.7%), *suppressor of others against decapentaplegic* (*SMAD4*) (6.5%), and *PIK3CA* (6.0%). Guo et al. [19] investigated 899 patients with ICC and found that *TP53* (18–40%) and *KRAS* (10–18%) were high-frequency mutation genes. Other mutations were found in *ARID1A*, *SMAD4*, *spectrin repeat-containing nuclear envelope protein 1* (*SYNE1*), *mucin 16* (*MUC16*), *BAP1*, *LDL receptor-related protein 1B* (*LRP1B*), *fibrous sheath-interacting protein 2* (*FSIP2*), *ephrin type-A receptor 2* (*EPHA2*), *IDH1*, *IDH2*, *polybromo 1* (*PBRM1*), *v-raf murine sarcoma viral oncogene homolog B1* (*BRAF*), *ATM*, *FGFR2*, *C16orf3*, *human leukocyte antigen A* (*HLA-A*), *HLA-C*, *titin gene* (*TTN*), *family with sequence similarity 230 member A* (*FAM230A*), *AHNAK2*, and *CTD-3193O13.9.* The most frequent fusion was observed in *FGFR2* (4.7%), and the highest amplification rates were in *human epidermal growth factor receptor 2* (*Her2/neu*) (4.7%), *myelocytomatosis oncogene* (*MYC*) (3.2%), *murine double minute 2* (*MDM2*) (3.2%), *mesenchymal-epithelial transition factor* (*cMET*) (2.3%), *cyclin D1* (*CCND1*) (2.0%), and *Cyclin E1* (*CCNE1*) (2.0%) [16] (see Table 1).

Predictive markers of immune checkpoint blockade are identified in 13% of the ICC tumors, and the most commonly found is *programmed cell death ligand 1* (*PD-L1*) overexpression, seen in 8%. Increased *microsatellite instability* (*MSI-H*) is seen in only a minority of the patients [16].

Comparing ICC with gall bladder cancer, ICC has significantly higher *IDH1* (14.5%), *BAP1* (9.5%), and *PBRM1* (7.5%) mutation rates. On the other hand, ICC has lower mutation rates of *TP53*, *SMAD4*, *APC*, and *ERBB2* and significantly less frequent mutations in *KRAS*, *CDKN2A*, and *BRCA1* than in extrahepatic cholangiocarcinoma. *Her2* overexpression and amplification are more associated with gallbladder cholangiocarcinoma (9–14%) and extrahepatic cholangiocarcinoma (4–8%) than with ICC (1%) [16]. *FGFR2* fusions are seen most often in ICC. *BRAF* mutations are infrequent in biliary tract cancers and almost exclusively found in ICC [20]. *C-MET* expression is associated more commonly with extrahepatic cholangiocarcinoma than with ICC [21]. The presence of immune checkpoint inhibitor-associated biomarkers is more common in ICC than in extrahepatic cholangiocarcinoma [16].

**Figure 1:**
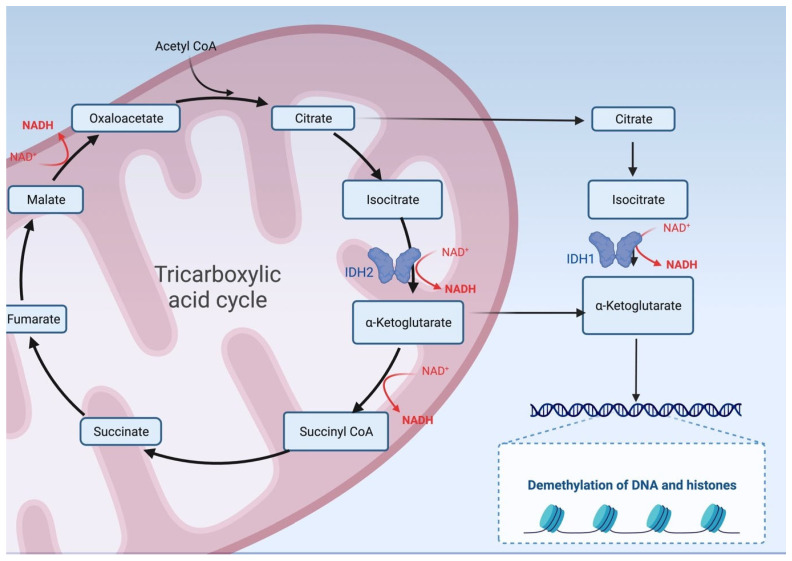
Isocitrate dehydrogenase (IDH) is an enzyme that plays a critical role in cellular metabolism, specifically in the tricarboxylic acid cycle. IDH catalyzes the conversion of isocitrate into alpha-ketoglutarate. IDH mutations promote the accumulation of 2-hydroxyglutarate, preventing the demethylation of DNA and histones and promoting cancer initiation.

Isocitrate dehydrogenase (IDH) 1 and 2 mutations lead to alterations in cellular metabolism and can lead to the production of oncometabolites and epigenetic changes [21]. IDH mutations are almost exclusively found in ICC. IDH is an enzyme that plays a critical role in cellular metabolism, specifically in the tricarboxylic acid (TCA) cycle. The TCA cycle generates adenosine triphosphate (ATP), a molecule that provides energy for various cellular processes. IDH catalyzes the conversion of isocitrate, a TCA cycle intermediate, into alpha-ketoglutarate (α-KG) while reducing nicotinamide adenine dinucleotide phosphate (NADP+) to NADPH in the process. *IDH1* and *IDH2* play a central role in cellular metabolism, lipid synthesis, and cellular defense against oxygen-free radicals [23]. IDH mutations promote the accumulation of 2-hydroxyglutarate (2-HG), which may promote cancer initiation [24] (see Figure 1).

Fusion events involving the fibroblast growth factor receptor (*FGFR*) gene are observed in a subset of ICC cases, mainly the *FGRR2*. *FGFR2* fusions have numerous partners, which may be a strong consideration for NGS testing, allowing identification of all fusions, including those less frequent. The *FGFR* family is composed of four tyrosine kinase receptors (*FGFR1*, *FGFR2*, *FGFR3*, and *FGFR4*) [25]. *FGFR* is a receptor tyrosine kinase that participates in cell growth, differentiation, and tissue repair. Upon interaction with growth factors belonging to the fibroblast growth factor (FGF) family, *FGFRs* undergo dimerization, activating intracellular signaling pathways that play a crucial role in stimulating cell proliferation and supporting cell survival [26].

Alterations in the *TP53* gene play a pivotal role in the development and progression of cancer. *TP53*, often called the “guardian of the genome”, encodes the p53 protein, a transcription factor and critical regulator of cell cycle control, DNA repair, and apoptosis [27]. The normal function of p53 is to act as a tumor suppressor. It monitors DNA integrity and, in response to DNA damage or other stress signals, influences the cell cycle to allow for DNA repair or initiates apoptosis if the damage is irreparable. *MDM2* protein binds to p53 tightly, inhibits p53 activity, and prevents p53 degradation [28]. The DNA damage response signaling induces activation of p53 and MDM2, and finally, activation of p53 target genes, which promote DNA repair, altered metabolism, or cell death [29]. Mutations or alterations in the *TP53* gene can result in the loss of these tumor-suppressing functions. This allows damaged cells to continue dividing, potentially accumulating additional mutations and leading to cancer development. *TP53* mutations can contribute to genetic instability within cancer cells. When p53 is dysfunctional, cells may not correctly repair DNA damage, leading to an increased likelihood of acquiring further genetic alterations. In an animal model, Hill et al. [30] investigated the consequences of the *TP53* mutation in hepatocytes and cholangiocytes. The authors found evidence that *TP53* loss promotes the reprogramming of hepatocytes to cholangiocytes, facilitating the formation of hepatocyte-derived ICC.

**Figure 2:**
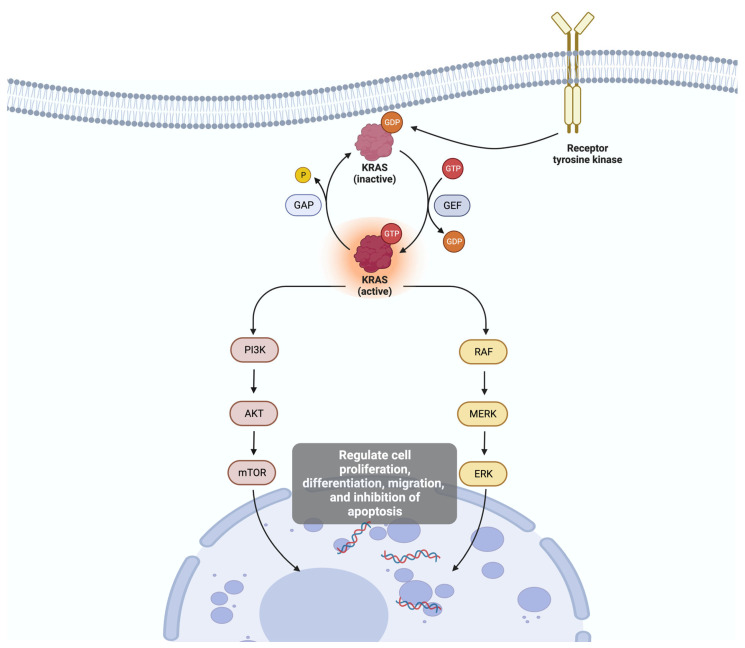
In its normal state, the Kirsten rat sarcoma virus (KRAS) protein acts as a molecular switch, cycling between active (GTP-bound) and inactive (GDP-bound) forms to transmit signals for cell survival and proliferation in response to external growth signals. Activated *KRAS* protein can act on the *RAF-MEK-ERK* and the *PI3K-AKT-mTOR* pathways, which regulate cell proliferation, differentiation, migration, and inhibition of apoptosis.

*KRAS* is a proto-oncogene member of the rat sarcoma viral oncogene family (*RAS*), and it is responsible for multiple cell signaling pathways. In its normal state, the *KRAS* protein acts as a molecular switch, cycling between active (GTP-bound) and inactive (GDP-bound) forms to transmit signals for cell survival and proliferation in response to external growth signals. Activated *KRAS* protein can act on the *RAF-MEK-ERK* and the *PI3K-AKT-mTOR* pathways, which regulate cell proliferation, differentiation, migration, and inhibition of apoptosis [31,32] (see Figure 2). When mutated, *KRAS* can become an oncogene, driving uncontrolled cell growth and contributing to the development and progression of cancer. Mutations in the *KRAS* gene lead to a permanently active form of the protein, causing continuous signaling and unregulated cell growth and division. This constitutive signaling is a hallmark of *KRAS*-mutated cancers [33].

**Figure 3:**
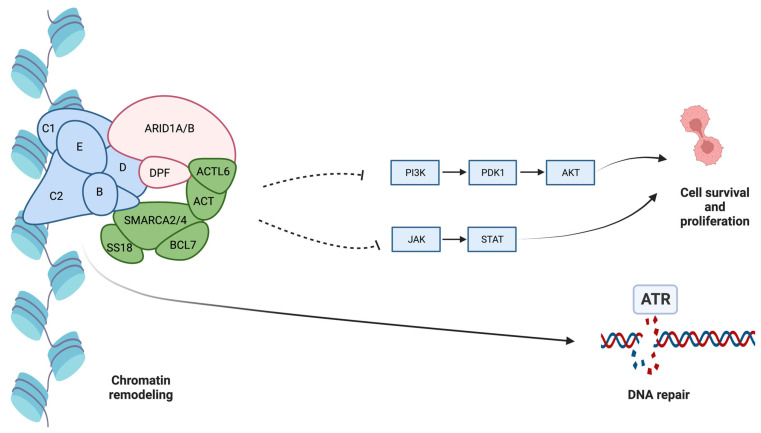
AT-rich interaction domain 1A (ARID1A) is a part of the SWI/SNF chromatin remodeling complex. ARID1A inhibits the PI3K/AKT and JAK/STAT pathways, limiting cell survival and proliferation capability. Additionally, ARID1A promotes DNA repair, avoiding the accumulation of mutations. Dotting lines represent inhibition while solid lines represent stimulation.

*ARID1A* (AT-rich interaction domain 1A) is a tumor suppressor gene that participates in chromatin remodeling and gene regulation. *ARID1A* is a part of the *SWI/SNF* chromatin remodeling complex responsible for binding DNA, controlling gene expression patterns, and DNA damage response. Mutations in *ARID1A* can disrupt the function of *PI3K/AKT/mTOR*, DNA damage, *EZH2*, and other signals. Mutations in the *ARID1A* gene can impair tumor-suppressing functions, allowing cells to divide and proliferate uncontrollably [34]. *ARID1A* knockout cells lose significant capacity to induce primary repairing double-strand breaks (DSBs) in DNA despite sparing DSB repair through poly(ADP-ribose) polymerase (PARP) pathways. The inhibition of PARP promotes a cytotoxic effect in *ARID1A* knockout cells [35]. *ARID1A* loss leads to ataxia telangiectasia and Rad3-related protein (ATR) activation signaling due to DNA damage [36]. Inhibition of ATR promotes cell death (see Figure 3). Additionally, *ARID1A* loss may also interfere with the immune checkpoint, promoting *MLH1* silencing and upregulation of *PD-L1* (programmed death ligand 1), leading to cancer cells escaping from immune checkpoint surveillance [37].

Xue et al. [87], at the cBioPortal, studied mixed and combined hepatocellular carcinoma (HCC) and ICC. The authors found that combined-type and mixed-type tumors have a distinct molecular and clinical profile and therefore are distinct subtypes. The combined-type demonstrated more ICC-like features, such as higher expression of *EPCAM*, *KRT19*, and *PRDM5*, as well as enrichment of *KRAS* mutations. On the other hand, mixed-type showed more HCC-like features, such as higher expression levels of *AFP*, *GPC3*, *APOE*, *and SALL4.* They indicated that therapies for ICC may better suit combined-type cHCC–ICC, whereas therapies for HCC may be adopted to treat mixed-type cHCC–ICC patients. They also found that the neuroepithelial stem cell protein, also known as Nestin, was more frequent in mixed tumors. The gene mutation related to Nestin expression might have a significant role in the carcinogenesis of ICC and hepatocellular carcinoma mixed tumors and can serve as a biomarker for the diagnosis and prognosis of combined hepatocellular–ICC.

### 3.2. Regional Variations in Molecular Patterns

*ERBB2* and *TP53* mutations are more associated with *Opisthorchis viverrini* and *Clonorchis cinensis*, which are more common in Southeast Asia, and endemic liver fluke infections [38]. Chronic inflammation caused by these parasites contributes to *TP53* mutation-driven tumorigenesis. *ARID1A* mutations are also more common in liver fluke-related cholangiocarcinoma [39]. Viral hepatitis-positive cholangiocarcinoma also has a higher frequency of *TP53* mutations [40].

*PIK3CA* mutations are rare in ICC [41]. However, in a study of Asian patients [42], *PIK3CA* mutations were identified in 32.4%, suggesting that *PIK3CA* mutations may be associated with significant dependence on regional geography. *Her2* mutations are equally infrequent in Western and Asian populations [43].

### 3.3. Tumor Characteristics According to Molecular Subtypes

Molecular profiling and genetic mutations play a pivotal role in shaping the tumor characteristics of ICC. Understanding the correlation between ICC’s molecular alterations and individual patient features is crucial for accurate diagnosis, treatment planning, and prognostication.

Sadot et al. [44] retrospectively studied computed tomography images of ICC and classified them by qualitative features obtained before surgery. Biopsies were taken concurrently from the tumor and non-tumor liver. The texture feature was significantly associated with *VEGF* expression. Correlation and entropy texture features were significantly related to *EGFR* expression. There were no significant associations between the texture features of *HIF-1α*, *CA-IX*, *p53*, *MRP-1*, *MDM2*, *CD24*, or *GLUT1*.

Kipp et al. [22] evaluated 94 formalin-fixed, paraffin-embedded cholangiocarcinomas (intra and extrahepatic). *IDH* mutations were more frequently found in tumors with evident cell changes and poor differentiation grades. Age, sex, tumor volume, and grade of cellular differentiation were not associated with *IDH* mutants. The *IDH* mutant type seems more associated with higher CA-19-9 levels, and patients with ICC and *FGFR2* fusions are younger than those without these alterations [45].

Farshidfar et al. [88] in The Cancer Genome Atlas (TCGA) performed a genomic analysis, evaluating 38 cholangiocarcinomas, predominantly from North America. The authors identified a distinct subtype enriched for IDH mutants. The IDH mutant-enriched subtype had high mitochondrial (especially components of the citric acid cycle and electron transport chain) and low chromatin modifier gene expressions, distinct mRNA and DNA methylation features, and low ARID1A expression. They also found that other IDH mutant liver cancers (such as HCC) demonstrated multiplatform similarities to ICC. Jiao et al. [89] found through exomic sequencing of 32 ICCs frequent inactivating mutations in multiple chromatin-remodeling genes (including BAP1, ARID1A, and PBRM1), as well as IDH1 and IDH2.

Boberg et al. [46] evaluated the molecular profile of cholangiocarcinoma arising in primary sclerosing cholangitis (PSC). The authors found that almost half of their cohort had mutations on *TP53* or *KRAS*, and 29% had mutations on *Ki-67*, suggesting these biomarkers have a central role in carcinogenesis in PSC.

Sasaki et al. [47] investigated the association between the *ARID1A* mutation and the histologic features of cholangiocarcinoma. The *ARID1A* mutation was more frequent in cholangiocarcinoma than mixed-tumor cholangio/hepatocellular carcinoma and was associated with ductal plate malformation patterns, which are developmental anomalies that originate from insults to the ductal plate.

*C-MET* expression in ICC is associated with microscopic cholangitis and mucus levels in tumors [48]. Additionally, the patients with *C-MET* overexpression were younger and more non-mass-forming than patients with normal or low *C-MET* expression [48].

Zou et al. [40] evaluated 103 patients with cholangiocarcinoma in China and found that *TP53*-defective ICC patients are more likely to be HBsAg-seropositive, whereas mutations in the oncogene *KRAS* are nearly exclusively found in HBsAg-seronegative ICC patients. They also found three pathways that are substantially affected in ICC: Ras/phosphatidylinositol-4,5-bisphosphate 3-kinase signaling, p53/cell cycle signaling, and transforming growth factor-β/Smad signaling.

### 3.4. Prognostication

Beyond guiding therapy, molecular profiling provides valuable prognostic information. Certain genetic signatures and biomarkers discovered through profiling can help predict disease aggressiveness and patient outcomes. This prognostic insight aids healthcare professionals in making informed decisions about treatment and follow-up care. Table 2 summarizes the main prognostic implications of the main mutations in ICC.

*KRAS* and *TP53* mutations are associated with increased tumor mutational burden, and consequently, they are associated with a poor prognosis in ICC patients, with dismal survival rates [19]. *MDM2*, the main p53-interactive protein, is also correlated with poor survival rates and advanced oncologic stages in cholangiocarcinoma [49].

Overexpression of the *MET* oncogene is an independent predictor of poor overall and disease-free survival rates in patients with cholangiocarcinoma [21]. Pu et al. [48] analyzed the expression of the *C-MET* gene by FISH and IHC. The authors found that *C-MET* overexpression was associated with the advanced oncological stage, mainly the T stage.

*ARID1A* alterations in cholangiocarcinoma are associated with poorer survival, a higher risk for vein invasion, and a higher risk for systemic and local recurrence [50,51]. Bi et al. [52] found that *ARID1A* alteration was associated with recurrence (HR = 1.71), and patients with Beclin-1 expression had a higher risk for mortality (HR = 2.39). The authors found no significant association between *IDH* and prognosis. Other authors also found that the *IDH* mutant has no significant association with either lymph node dissemination or overall or recurrence-free survival [45].

The *BRAF V600E* mutation in biliary tract cancer is associated with a higher oncologic stage, resistance to systemic chemotherapy, and a lower survival rate [53]. Additionally, *BRAF V600E* variants have a significant correlation with tumor volume, multinodular cancer, and vascular invasion [54]. ICC patients with *FGFR2* fusions have better overall and progression-free survival than those without *FGFR2* alterations [55]. *PD-L1* expression in biliary tumors is associated with prolonged progression-free survival (HR = 0.23) [56].

Jolissaint et al. [90] found that node-positive patients with at least one high-risk genetic alteration (*TP53* mutation, *KRAS* mutation, *CDKN2A/B* deletion) had worse survival compared to wild-type patients (median OS, 12.1 months; 95% CI, 5.7–21.5; *p* = 0.002), regardless of treatment.

Boerner et al. [91] found, in a bi-institutional study with 412 patients with ICC, that IDH1 was the most common oncogenetic alteration and that *TP53*, *KRAS*, and *CDKN2A* alterations were independent prognostic factors in iCCA when controlling for clinical and pathologic variables, disease stage, and treatment.

### 3.5. Clinical Applications in Precision Medicine

Molecular profiling enables the identification of actionable mutations and molecular targets within tumors, guiding the selection of targeted therapies that have the potential to be less toxic and more effective than traditional treatments.

#### 3.5.1. Predicting Treatment Response and Treatment Selection

Genetic testing is pivotal in predicting how individual ICC tumors may respond to different treatments. Genetic testing can reveal a tumor’s sensitivity or resistance to various chemotherapy agents. This information helps healthcare professionals select the most effective chemotherapy regimens and avoid the toxicity of treatments that are unlikely to benefit the patient. Molecular profiling enables the identification of specific genetic or protein expression alterations and biomarkers unique to individual ICC tumors. This information allows healthcare professionals to tailor treatment strategies with remarkable precision. Patients’ selection of certain treatment types helps save money on expensive and ineffective treatments, avoid unnecessary toxicity, and reduce time expenditure with exposure to treatments that will not help.

The high expression of certain markers, such as thymidylate synthase (*TS*), ribonucleotide reductase large subunit M1 (*RRM1*), and excision repair cross-complementation group 1 (*ERCC1*), can be associated with sensitivity or resistance to some chemotherapy regimens. These proteins can be mutated in over 25% of biliary tract cancer cases [16]. Patients with low *TS* expression have improved response rates to fluoropyrimidine-based chemotherapy compared to those with *TS* overexpression [57]. Cytoplasmic *RRM1* activation promotes gemcitabine resistance [58]. *ERCC1* is associated with resistance to platinum-based chemotherapy [59]. The mutant *TP53* is associated with chemoresistance to Gemcitabine [60].

#### 3.5.2. Targeted Therapies

Identifying actionable mutations can guide the selection of targeted therapies. These drugs inhibit specific molecular pathways driving ICC growth, potentially resulting in improved treatment responses and fewer side effects. The use of targeted therapy in ICC significantly improves patients’ survival compared with patients who received the traditional “one size fits all” therapy (HR: 2.06) [17].

*TP53* is one of the major focuses of target therapy since its mutation is one of the most frequent in several types of cancer. Acting on this mutation could be a game-changer for the prognosis of most cancer types. However, developing p53-target therapy is challenging because of the risk of toxicity by damaging the wild-type p53 while acting against mutant p53, potentially promoting cancer rather than inhibiting it. *TP53* has a wide range of functions beyond tumor suppression, including cellular metabolism and immune regulation. Targeting p53 could potentially disrupt these critical cellular processes, leading to undesirable side effects. Unlike some other proteins with well-defined drug binding sites, p53 lacks such druggable pockets. It has a relatively flat and featureless surface, making it challenging to design small molecules that can effectively bind and modulate p53’s activity [61].

*MDM2* can negatively regulate the stability and activity of wtp53. Several *MDM2* inhibitors have been studied in pre-clinical tests to act against malignant cells [61]. Nutlin-3 was the first drug to disrupt the interaction between p53 and *MDM2* [62]. In vivo and in vitro investigations demonstrated that SAR405838, a specific *MDM2* antagonist, induces activation of wtp53 [63].

Fiorini et al. [60] found that mutant p53 is associated with chemoresistance to Gemcitabine, one of the main standard treatment alternatives for cholangiocarcinomas [64]. In the next few years, the synergic effect of Eprenetapopt, a drug that restores the wild-type p53 in cells with mutant p53, and chemotherapic agents might improve survival by avoiding chemoresistance [65,66].

Similar to *TP53*, targeting *KRAS* has the potential to revolutionize cancer treatment due to the high frequency of *KRAS* mutations in overall cancers. However, targeting *KRAS* is quite challenging. There are multiple different *KRAS* mutations with varying properties, and *KRAS* has multiple conformations and can switch between them. Additionally, *KRAS* lacks easily druggable binding pockets, and targeting the active GTP-bound form of *KRAS* is particularly challenging because this form is transient and difficult to stabilize [67]. Currently, numerous studies targeting *KRAS* are being investigated in different stages of pre-clinical and clinical study, such as Sotorasib, Adagrasib, Selumetinib, and Trametinib, and future trials should determine the value of *KRAS* inhibitors in ICC [67]. Due to the difficulty of targeting *KRAS*, some drugs aim at the *KRAS* downstream effectors, such as Raf and *MEK1/2* proteins [68]. Dong et al. [69], in an ICC in vitro and in vivo study, assessed the potential of *MEK* inhibitors (U0126, PD901, and Selumetinib). The authors observed significant growth inhibition due to reduced proliferation and enhanced apoptosis.

Since *ARID1A* mutation cells induce upregulation of ATR, blocking ATR activity could work as an ICC treatment by reducing cancer cell survival. ATR inhibitors, including Berzosertib and Ceralasertib, can potentially act on *ARID1A*-mutant cancer [70]. Additionally, *ARID1A* knockout cells spare *PARP* pathways, and inhibition of *PARP*, such as Olaparib, Niraparib, Rucaparib, and Veliparib, could accelerate cancer cell death [37].

*IDH* mutations, mainly *IDH1* mutations, have a particularly relevant role in ICC due to their current potential in target therapy. *IDH* mutations are almost exclusively found in ICC among the cholangiocarcinomas. A multicentric phase III trial (ClarIDHy) investigated the role of Ivosidenib, a small-molecule inhibitor of mutated *IDH1*, in cholangiocarcinoma with *IDH1*-mutant and refractory to chemotherapy. The progression-free survival (2.7 vs. 1.4 months) [71] and the overall survival (10.3 vs. 7.5 months) [72] were higher in the Ivosidenib group than in the placebo group. *IDH2* mutations are rarer in ICC than in *IDH1*. Enasidenib is an *IDH2* inhibitor, and it is currently under investigation in a phase I/II trial for solid tumors, including cholangiocarcinoma (NCT02273739).

*FGFR2* fusions and rearrangements are other relevant potential targets in patients with ICC. A multicentric phase I/II open-label trial investigated the use of Derazantinib in advanced or unresectable ICC with *FGFR2* gene fusion [73]. Derazantinib is a pan-*FGFR* activity that is capable of inhibiting several kinases. The results were encouraging, showing a global response rate of 21% and a disease control rate of 83%. Another phase I/II trial investigated another *FGFR* inhibitor, Pemigatinib, and demonstrated a partial or complete response in 35.5% of patients with advanced cholangiocarcinoma [74]. Infigratinib is an *FGFR1–3*-selective inhibitor and showed a 23% objective response rate in an open-label single-arm trial [75]. Other potential *FGFR* inhibitors for ICC include Futibatinib, Ponatinib, Erdafitinib, and Debio 1347 [76].

Currently, there are numerous FDA-approved *Her2*-targeted drugs, including monoclonal antibodies (Trastuzumab and Pertuzumab) and small-molecule tyrosine kinase inhibitors (Lapatinib, Tucatinib, Neratinib, and Varlitinib) [77]. Additionally, *PI3K* inhibitors like Taselisib might also work for *Her2* overexpression cancer cells, considering that *HER2* induces the *PI3K/AKT/mTOR* pathway [78]. However, *Her2* overexpression and amplification are less common in ICC than in gall bladder or extrahepatic cholangiocarcinoma, and consequently, only a minority of patients may benefit from *Her2*-target therapy [16]. A phase II trial (TreeTopp) evaluated Varlitinib (a poly-tyrosine kinase inhibitor, including *Her2*) plus capecitabine for advanced cholangiocarcinoma. The authors found no significant improvement in objective response, progression-free survival, or overall survival. However, the authors suggest that adding Varlitinib may improve progression-free survival in the female subgroup [79]. Javle et al. [80], in a multicentric open-label phase II study, evaluated the use of Pertuzumab and Trastuzumab for *Her2*-positive stage IV cholangiocarcinoma (MyPathway). The authors found an objective response rate of 23%.

Ring finger protein 43 (*RNF43*) mutations are found in less than 2% of ICC [14]. *RNF43* regulates the Wnt signaling pathway. WNT974 is a potent inhibitor of Porcupine, which interacts with *Wnt* and may enhance checkpoint inhibitor activity and, consequently, may have a role in a small percentage of ICC patients [81].

A phase II single-arm study evaluated *BRAF* and *MEK* inhibitors (Dabrafenib and Trametinib, respectively) for cholangiocarcinoma with a *BRAF V600E* mutation [82]. The authors found that a 51% rate of investigator-assessed overall response was achieved. A study using drug screens in patient-derived organoids with different *BRAF* variants showed encouraging results with *BRAF* inhibitors in patients with advanced cholangiocarcinoma [54].

#### 3.5.3. Immunotherapy

Molecular profiling may uncover immune-related biomarkers, informing the use of immunotherapy agents like immune checkpoint inhibitors. These drugs can enhance the immune system’s ability to recognize and attack ICC cells, offering promising avenues for treatment. However, only a few ICC patients may benefit from immune checkpoint blockade. Only 8% of patients overexpress *PD-L1*, and 2% express *MSI-H* [16]. The presence of immune checkpoint inhibitor-associated biomarkers is more common in ICC than in extrahepatic cholangiocarcinoma.

The *ARID1A* deficiency interferes with the immune checkpoint and predicts microsatellite instability and overexpression of *PD-L1* [83]. Consequently, *PD-L1* inhibitors might have a promising role in *ARID1A*-mutant ICC.

Pembrolizumab, Nivolumab, and Durvalumab are anti-*PD-1* antibodies. A recently published double-blind phase III trial (KEYNOTE-966) compared Pembrolizumab with placebo for unresectable, locally advanced, or metastatic biliary tract cancer. Both groups received Gemcitabine and Cisplatin. The authors found that the overall survival was higher in the Pembrolizumab group (12.7 vs. 10.9 months) [84].

A multicentric phase II trial investigated Nivolumab for advanced refractory biliary tract cancer [56]. The study was not controlled. The authors found a response rate of 22%, detected by radiological imaging.

#### 3.5.4. Monitoring Treatment Responses

Continual genetic testing during treatment can monitor tumor molecular profile changes. This real-time information enables oncologists to adapt treatment plans as needed. If a patient develops resistance to targeted therapy, post-progression biopsies with molecular testing can reveal new mutations that guide the selection of alternative treatments.

Multiple mechanisms contribute to the acquisition of resistance. The outgrowth of *RTK* pathway mutations and *2-HG*-restoring mutations seems to have a significant role in targeted mutant *IDH1* inhibitors [85]. Other mutations in other genes, like *TP53*, *ARID1A*, or *PIK3R*, or even in a second-site mutation in *IDH*, can also contribute to drug resistance.

Goyal et al. [86] reported acquired resistance to Infigratinib, an *FGFR* inhibitor, in three patients with *FGFR2* fusion-positive ICC. A biopsy of post-progression tumors and autopsies showed significant tumor heterogeneity and different *FGFR2*-acquired mutations, which explained the resistance to Infigratinib.

## 4. Discussion

### 4.1. Current Literature Gaps

While molecular profiling and genetic analysis hold immense promise for improving our comprehension and treatment of ICC, it is essential to acknowledge the existing gaps and limitations within the literature. One of the primary limitations of ICC molecular profiling is the scarcity of comprehensive datasets. ICC is a relatively rare cancer, and obtaining a sufficiently large sample of patients with detailed molecular profiling data can be challenging. Consequently, studies may be underpowered, limiting their ability to draw robust conclusions regarding the significance of specific genetic alterations or molecular subtypes.

ICC tumors frequently present significant intratumoral heterogeneity. A single tumor can harbor various genetic mutations and molecular alterations, making it challenging to pinpoint a single target for therapy. This heterogeneity also poses difficulties in selecting appropriate samples for molecular analysis, as a single biopsy may not capture the full genetic diversity of the tumor. Standardization of molecular profiling and genetic testing protocols is crucial for ensuring consistency and comparability of results across different research studies and clinical settings. Variability in testing methods and data analysis can lead to discrepancies and hinder the validation of findings.

While molecular profiling can identify potential therapeutic targets, access to targeted therapies remains challenging. Regulatory approvals, insurance coverage, and cost considerations often limit these treatments. Consequently, even when actionable mutations are identified, patients may face barriers to receiving targeted treatments. The widespread adoption of ICC molecular profiling faces several challenges. The high cost of access to some target drugs may increase the gap between rich and poor countries. Addressing these challenges is essential to harnessing the full potential of molecular profiling in ICC and making it accessible to all patients. Future studies should investigate strategies on how to spread precision medicine worldwide.

### 4.2. Future Prospects

The future of molecular profiling in ICC holds great promise. Advancements in technology, including more cost-effective sequencing methods and improved data analysis tools, will likely refine our understanding of ICC’s molecular landscape. As our knowledge deepens, new therapeutic targets may emerge, paving the way for developing novel drugs specifically tailored to ICC’s unique molecular subtypes. The integration of liquid biopsies, which allow for non-invasive monitoring of molecular changes, could also revolutionize how ICC is managed, enabling dynamic adjustments to treatment plans based on real-time molecular data.

Currently, most target therapies are advised as second- or third-line therapies for advanced or unresectable ICC. However, future studies should investigate the role of these therapies as neoadjuvant or adjuvant therapies for patients treated with curative intention.

## 5. Conclusions

Molecular profiling in ICC represents a groundbreaking approach to understanding and managing this complex liver cancer. As our comprehension of ICC’s molecular intricacies continues to expand, so does the potential for offering patients more precise and effective treatments. The integration of molecular profiling into clinical practice signifies the dawn of a new era in ICC care, emphasizing personalized medicine in the ongoing battle against this malignancy.

## Figures and Tables

**Table 1:** Some of the main mutations found in intrahepatic cholangiocarcinoma and their corresponding target therapy options.

Mutation	Frequency (%) *	Potential Target-Therapies
*TP53*	18–43	Idasanutlin, Alrizomadlin, Eprenetapopt, SAR405838, Milademetan
*IDH1*	22	Ivosidenib
*IDH2*	5	Enasidenib
*KRAS*	10–18	Sotorasib, Adagrasib, Selumetinib, Trametinib
*ARID1A*	19–22	Olaparib, Niraparib, Rucaparib, Veliparib, Ceralasertib, Berzosertib
*BAP1*	9–19	Olaparib
*BRAF*	7	Dabrafenib, Trametinib, Vemurafenib, Encorafenib
*FGFR2*	5	Derazantinib, Infigratinib, Pemigatinib, Futibatinib, Ponatinib, Erdafitinib, Debio 1347
*Her2*	1	Trastuzumab, Pertuzumab, Lapatinib, Tucatinib, Neratinib, Varlitinib, Taselisib

* According to Guo et al. and Weinberg et al. [16,19]. *TP53*: *tumor protein p53*; *IDH1*: *isocitrate dehydrogenase 1*; *IDH2*: *isocitrate dehydrogenase 2*; *KRAS*: *Kirsten rat sarcoma virus*; *ARID1A*: *AT-rich interactive domain-containing protein 1A*; *BAP1*: *BRCA1-associated protein-1*; *BRAF*: *V-raf murine sarcoma viral oncogene homolog B*; *FGFR2*: *fibroblast growth factor receptor 2*; *Her2*: *human epidermal growth factor receptor 2*.

**Table 2:** Prognostic implications of the main mutations in ICC.

Mutation	Prognostic Implications
*TP53*	Advanced stages, increased tumor mutational burden, and poor survival rates
*IDH1*	No significant association with either lymph node dissemination or overall or recurrence-free survival
*IDH2*	
*KRAS*	Increased tumor mutational burden and poor survival rates
*ARID1A*	Poor survival, higher risk for vein invasion, and higher risk for recurrence (systemic or local)
*C-MET*	Advanced oncological stage, mainly the T stage
*BRAF*	Higher oncologic stage, resistance to systemic chemotherapy, and lower survival rate
*FGFR2*	Better overall and progression-free survival

*TP53*: *tumor protein p53*; *IDH1*: *isocitrate dehydrogenase 1*; *IDH2*: *isocitrate dehydrogenase 2*; *KRAS*: *Kirsten rat sarcoma virus; ARID1A*: *AT-rich interactive domain-containing protein 1A*; *C-MET*: *hepatocyte growth factor receptor*; *BRAF*: *V-raf murine sarcoma viral oncogene homolog B*; *FGFR2*: *fibroblast growth factor receptor 2*.

## Data Availability

No new data were created.
